# A171 EXPLORING PATIENT PERSPECTIVES ON A 12-WEEK, ONLINE STRESS REDUCTION INTERVENTION IN INFLAMMATORY BOWEL DISEASE

**DOI:** 10.1093/jcag/gwab049.170

**Published:** 2022-02-21

**Authors:** A Hyde, M Watt, F Peerani, K Madsen, J Siffledeen, K Kroeker, A Lim, P Tandon

**Affiliations:** University of Alberta Faculty of Medicine & Dentistry, Edmonton, AB, Canada

## Abstract

**Background:**

Inflammatory bowel disease (IBD) which includes Crohn’s disease (CD) and ulcerative colitis (UC), is a chronic relapsing-remitting or progressive inflammatory condition. In addition to physical symptoms, individuals with IBD experience uncertainty about their future, social isolation, and increased psychological stress. Conventional medical and surgical IBD treatment does not adequately address the the impact of psychological stress. Online stress reduction interventions may therefore be useful adjuncts to standard medical therapies for IBD. The Peace Power Pack (PPP) trial was a 12-week RCT testing a stress reduction intervention with two core components: (i) a yoga, breathwork and meditation video, and (ii) a behavior-change facilitation informed by cognitive behavioral therapy.

**Aims:**

The aims of this qualitative study, which was carried out alongside the PPP trial was to: (i) explore the experience of living with IBD, (ii) understand the impacts of the PPP program, and (iii) identify potential improvements to the program.

**Methods:**

Upon completion of the 12-week RCT, all intervention participants were invited to participate in semi-structured interviews. A qualitative descriptive approach was used. Interviews were analyzed through a theoretical thematic analysis process, whereby transcripts were coded, and codes then grouped into larger categories and themes.

**Results:**

Of the 56 participants interviewed, 52% had CD and 48% had UC. Participants ranged in age from 23 to 73 years, were predominantly female (70%) and had been diagnosed with IBD 14.3 years ago. Three main themes (Table 1) were identified: (i) IBD as a source of stress and uncertainty, (ii) understanding the positive impacts of the stress reduction program, and (iii) suggested strategies to enhance program desirability. IBD was described as causing uncertainty, significant disruptions to daily activities, and stress. The online program was associated with a perceived reduction in IBD symptom burden, an increased ability to manage daily and disease-associated stressors, and a more positive mindset. Variation in program content and fostering connections with others in the IBD community were identified as potential strategies to enhance future programming.

**Conclusions:**

This study highlights the power of the patient voice to deepen our understanding of the impact of IBD, and the potential benefit of an online stress reduction program including suggestions for iterative refinement.

Table 1. Themes identified with sample quotations

**Funding Agencies:**

University of Alberta Hospital Foundation, the American College of Gastroenterology and the Inflammation, Microbiome, and Alimentation: Gastrointestinal and Neuropsychiatric Effects (IMAGINE) Network CIHR grant

**Table:** 

Theme	Sample quote
IBD as a sources of stress & uncertainty	“It’s stress from the fact that no one really knows the cause. No one really knows the cure and it’s maybe going to get worse over time”.
Understanding the positive impacts of the PPP program	I catch myself . . . I’m much more mindful of what’s going on stress wise . . . it changed how I look at stress and how I cope with it.
Enhancing program desirability	I wanted to talk to other people in the program who had similar life situations to me. Like working full-time, having young children at home . . . to share strategies they were using to make time to do this.

